# Lysosomes in Ferroptosis: Regulatory Mechanisms and Molecular Targets

**DOI:** 10.3390/molecules31132373

**Published:** 2026-07-06

**Authors:** Tingrui Luo, Chenyu Wang, Nanhao Zhou, Yuansheng Zhang, Xianbo Mou

**Affiliations:** Health Science Center, Ningbo University, Ningbo 315211, China; 226002066@nbu.edu.cn (T.L.); 226002096@nbu.edu.cn (C.W.); 226002158@nbu.edu.cn (N.Z.); 226002142@nbu.edu.cn (Y.Z.)

**Keywords:** ferroptosis, lysosomes, iron metabolism, autophagy, oxidative stress

## Abstract

Ferroptosis is a regulated form of cell death characterized by iron-dependent lipid peroxidation and membrane damage, with broad relevance to human disease. Accumulating evidence suggests that ferroptosis is governed by coordinated organelle-level regulation, among which lysosomes have emerged as central hubs. By controlling endolysosomal iron processing, transport, and degradation pathways, lysosomes shape the intracellular distribution and reactivity of iron, thereby modulating iron-driven lipid peroxidation. The acidic, iron-rich microenvironment and limited local antioxidant capacity render lysosomal membranes highly susceptible to oxidative injury, positioning lysosomes as initiation and amplification sites of lipid peroxidation. Meanwhile, lysosome-dependent selective autophagy pathways actively remodel iron homeostasis, lipid metabolism, and cellular antioxidant defenses, thereby dynamically modulating ferroptotic sensitivity. Mitochondria–lysosome crosstalk further redistributes iron, reactive oxygen species, and lipid substrates, linking lysosomal activity to interorganelle control of ferroptosis. Lysosomal stress-responsive signaling also coordinates metabolic adaptation and redox control. This review summarizes and integrates current evidence on lysosome-centered mechanisms that organize iron metabolism, lipid peroxidation, selective autophagy, organelle crosstalk, and stress-responsive signaling during ferroptosis, and further discusses their disease-specific roles, therapeutic potential, and translational challenges.

## 1. Introduction

Ferroptosis has emerged as a distinct form of regulated cell death, fundamentally different from apoptosis and necroptosis, and is characterized by iron-dependent lipid peroxidation and catastrophic membrane damage [1,2]. Since its initial identification, ferroptosis has been implicated in the pathogenesis of a broad spectrum of human diseases, including multiple types of cancer, neurodegenerative disorders, cardiovascular and ischemia–reperfusion injuries, as well as renal, hepatic, metabolic, and inflammatory conditions [3]. At the molecular level, ferroptosis is driven by dysregulated iron metabolism, excessive accumulation of lipid peroxides, and failure of intracellular antioxidant defense systems [4]. This cascade ultimately results in the generation and accumulation of phospholipid hydroperoxides (PLOOHs), which execute ferroptosis by triggering oxidative chain reactions that rapidly compromise membrane stability and cellular viability [5].

Early investigations of ferroptosis primarily focused on cytosolic redox regulation and mitochondrial metabolism, emphasizing the central roles of glutathione peroxidase 4 (GPX4), the System Xc−glutathione (GSH) axis, mitochondrial reactive oxygen species (ROS) generation, and iron–sulfur cluster. For many years, studies exploring organelle involvement in ferroptosis predominantly centered on mitochondria, which were considered key regulators of ferroptotic susceptibility through their roles in iron utilization, redox metabolism, and lipid peroxide generation [6]. However, accumulating evidence now indicates that isolated biochemical reactions do not govern ferroptosis but instead emerge from coordinated metabolic and signaling interactions among multiple subcellular organelles [7].

Among these organelles, lysosomes have recently gained recognition as critical regulators of ferroptotic signaling. Traditionally viewed as terminal degradative compartments, lysosomes are now understood as dynamic metabolic and signaling hubs that integrate intracellular degradation with nutrient sensing, autophagy regulation, and cellular stress responses to maintain cellular homeostasis [8]. Through these multifaceted functions, lysosomes occupy a central position at the intersection of iron metabolism, lipid turnover, and redox control—three core processes that define ferroptotic vulnerability.

Lysosomes regulate ferroptosis through multiple coordinated mechanisms, among which iron metabolism plays an important regulatory role. Through endocytic and autophagic pathways, lysosomes regulate intracellular iron trafficking by mediating endosomal iron release and ferritin degradation, thereby controlling the cellular pool of redox-active iron [9]. Lysosomes serve as the primary intracellular site for iron processing. Following transferrin receptor 1 (TfR1)-mediated endocytosis, transferrin (Tf)-bound ferric iron (Fe^3+^) is delivered to late endosomes and lysosomes, where the acidic microenvironment enables ferrireductase six-transmembrane epithelial antigen of prostate 3 (STEAP3) to promote its reduction to redox-active ferrous iron (Fe^2+^). The resulting Fe^2+^ is transported into the cytoplasmic labile iron pool by divalent metal transporter 1 (DMT1), supplying the redox-active iron that fuels Fenton reaction and lipid peroxidation [10]. In parallel, nuclear receptor coactivator 4 (NCOA4)-mediated ferritinophagy transports ferritin to lysosomes for degradation, releasing redox-active iron into the labile iron pool that drives Fenton reactions and exacerbates lipid peroxidation, thereby promoting ferroptosis [11,12,13]. In this context, lysosomes function not merely as passive degradative compartments but as active determinants of ferroptotic iron availability and sensitivity.

Beyond iron metabolism, lysosomes exert multifaceted control over ferroptotic susceptibility by regulating lipid peroxidation and antioxidant defense systems. Lysosomal acidification is critical for cystine deprivation-induced lipid peroxidation and ferroptosis, as an acidic lysosomal microenvironment supports the generation and distribution of redox-active iron and lipid radicals that drive membrane oxidative damage, whereas lysosomal alkalinization impairs this pathway and confers ferroptosis resistance [14]. Lysosomal lipid peroxidation can further induce lysosomal membrane permeabilization, leading to the release of Fe^2+^ into the cytosol and the amplification of cell-wide lipid peroxidation cascades [15]. Conversely, lysosome-mediated degradation of extracellular cysteine-rich proteins provides an alternative source of intracellular cysteine, sustaining glutathione synthesis and preserving GPX4 activity, thereby conferring resistance to ferroptotic stress under nutrient-limited conditions [16].

Lysosome-dependent autophagic pathways further establish a direct mechanistic link between lysosomal activity and ferroptosis regulation. Selective autophagy processes, including NCOA4-mediated ferritinophagy and chaperone-mediated autophagy (CMA), regulate ferroptosis by directing lysosomal degradation of key substrates such as ferritin and GPX4, thereby modulating intracellular iron levels, lipid peroxidation, and redox balance [12,17,18]. Through these mechanisms, lysosomes reshape intracellular iron availability, lipid composition, and antioxidant capacity, thereby determining cellular sensitivity or resistance to ferroptotic stress.

In addition to their degradative functions, lysosomes act as stress-responsive signaling platforms. TFEB-centered signaling coordinates lysosomal biogenesis, autophagy, and antioxidant adaptation, thereby modulating cellular susceptibility to ferroptosis [19,20].

Despite the growing recognition of lysosomes as pivotal regulators of ferroptosis, existing research remains fragmented and pathway-specific, often examining isolated processes such as ferritinophagy, iron trafficking, or lipid peroxidation without integrating their collective contributions to iron metabolism, redox homeostasis, autophagic flux, and stress-responsive signaling across ferroptotic stages. This gap underscores the need for a comprehensive framework to synthesize the spatial and functional orchestration of lysosome-centered networks in ferroptotic cell death and to translate these insights into targeted therapies.

In this review, we summarize recent advances in lysosomal roles during ferroptosis regulation, beginning with an outline of ferroptosis’s core biochemical features and lysosomal functions relevant to iron and redox homeostasis, followed by a systematic discussion of key mechanisms—including iron trafficking, autophagy-dependent modulation, lipid peroxidation control, and signaling networks—and concluding with emerging molecular targets and therapeutic opportunities, ultimately positioning lysosomes as central orchestrators of ferroptotic execution.

## 2. Molecular Mechanisms of Ferroptosis

**Figure 1:**
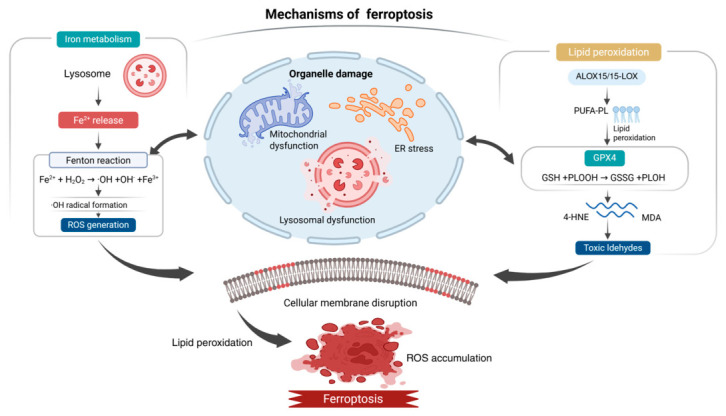
Mechanisms of ferroptosis. The left and central panels illustrate lysosomal iron release and organelle dysfunction, which enhance Fenton reactions, reactive oxygen species generation, and oxidative stress. The right panel highlights ALOX15-driven lipid peroxidation and impaired GPX4-dependent detoxification, culminating in toxic aldehyde accumulation and ferroptosis. ALOX15, arachidonate lipoxygenase 15; ER, endoplasmic reticulum; GPX4, glutathione peroxidase 4; GSH, glutathione; GSSG, glutathione disulfide; MDA, malondialdehyde; PUFA-PL, polyunsaturated fatty acid-containing phospholipids; ROS, reactive oxygen species; 4-HNE, 4-hydroxynonenal. Created in BioRender. Luo, T. (2026) https://BioRender.com/bff6g6i (accessed on 1 July 2026).

Iron-dependent lipid peroxidation is a defining feature of ferroptosis, characterized by the enzymatic oxidation of polyunsaturated fatty acid (PUFA)-containing phospholipids and the subsequent accumulation of PLOOH [2]. This process disrupts membrane fluidity, increases membrane permeability, and leads to organelle injury and ultimately cell death [1]. The dual catalytic role of iron in ferroptosis involves two primary mechanisms, as illustrated in Figure 1. The first involves the Fenton reaction, in which Fe^2+^ reacts with hydrogen peroxide (H_2_O_2_) to produce hydroxyl radicals (•OH) via Fe^2+^ + H_2_O_2_→•OH + OH− + Fe^3+^, thereby amplifying oxidative damage and initiating radical chain reactions within lipid bilayers [2,21]. The second mechanism is enzymatic catalysis, mediated by arachidonate lipoxygenase 15 (ALOX15)-dependent stereospecific peroxidation of arachidonoyl-phospholipids (C13-H abstraction), which requires an Fe^3+^-oxo center for completion of the catalytic cycle [22,23].

Lipid peroxidation, as an enzymatically driven process, is precisely regulated by the redox equilibrium between pro-oxidant pathways and antioxidant defense systems. Among these, the glutathione peroxidase 4 (GPX4) system constitutes a pivotal antioxidant barrier that detoxifies PLOOH according to the reaction: 2 GSH + PLOOH → GSSG + PLOH + H_2_O [24,25]. When antioxidant defenses are compromised (e.g., via pharmacological inhibition) or iron overload occurs, redox homeostasis is disrupted, leading to ROS accumulation [26,27]. These ROS preferentially peroxidize PUFA-containing phospholipids within cellular membranes, generating PLOOH and cytotoxic aldehydes (e.g., 4-hydroxynonenal (4-HNE) and malondialdehyde (MDA)) [28]. The subsequent membrane destabilization ultimately leads to organelle dysfunction and plasma membrane rupture, hallmarks of ferroptosis [29].

Emerging evidence positions lysosomes as a regulatory hub in ferroptotic cascades, acting through two interconnected axes: iron metabolism and lipid peroxidation modulation. This lysosomal dual functionality underscores the need for systematic investigation into their spatiotemporal dynamics during ferroptosis.

### 2.1. Lysosomal Structure and Function

As central effectors of ferroptotic pathways, lysosomes play a pivotal role in regulating cellular iron metabolism and maintaining redox homeostasis. Structurally, the lysosomal membrane establishes both a physical and biochemical barrier that protects cells from oxidative stress. The integrity of the lysosomal membrane is fundamental to its resistance to oxidative stress. Its membrane is enriched in cholesterol, a feature that enhances membrane stability and protects against oxidative damage [30]. Moreover, lysosome-associated membrane proteins (LAMPs) reinforce mechanical integrity, acting as structural shields against ROS-induced permeability. Suppression of LAMPs in lysosomes leads to increased sensitivity to lysosomal cell death (LCD) pathways [31]. Lysosomes contain over 60 acid hydrolases responsible for degrading a wide range of biomolecules, including proteins, nucleic acids, and invading pathogens, through heterophagic and autophagic pathways [32]. Their acidic lumen (pH 4.5–5.0) provides an optimal environment for these enzymes, ensuring efficient substrate degradation and protection against oxidative insults.

In addition to their structural resilience, lysosomes also play a crucial role in maintaining cellular iron homeostasis as a metabolic hub and repairing oxidative damage. Recent studies have highlighted the centrality of the lysosomal membrane in iron regulation. Through the concerted actions of lysosomal transmembrane proteins, they finely regulate iron uptake, storage, and release to sustain metabolic balance [33]. In addition, lysosomes mediate ferritin turnover via ferritinophagy, a selective autophagic process that maintains intracellular iron levels [12]. Growing evidence further implicates the endosomal sorting complexes required for transport (ESCRT) machinery in preserving lysosomal integrity under oxidative stress. Skowyra et al. demonstrated that ESCRT components are recruited to sites of lysosomal membrane disruption to facilitate repair. At the same time, Dai et al. revealed that this repair pathway protects cells from ferroptosis [34,35]. Collectively, these findings support a model in which ESCRT serves as a common effector at the lysosomal membrane, facilitating the repair of oxidative damage to prevent ferroptosis.

Lysosomes act as pivotal hubs in cellular adaptation to oxidative stress, integrating degradative and signaling functions. Through cooperation with the autophagic machinery, lysosomes selectively eliminate ROS-generating substrates, such as damaged mitochondria and peroxidized lipid droplets, thereby limiting oxidative injury [1,36,37]. Lysosomes also function as signaling platforms that coordinate cellular adaptation to oxidative stress. Activation of TFEB promotes lysosomal biogenesis and autophagy-related gene expression, thereby enhancing degradative capacity and stress adaptation [38,39,40]. Meanwhile, the nuclear factor erythroid 2-related factor 2 (NRF2)-Kelch-like ECH-associated protein 1 (KEAP1) signaling pathway regulates lysosomal biogenesis by activating the TFEB transcription factor [20].

Together, lysosomes protect cells from ROS-induced injury through coordinated structural, metabolic, and adaptive responses. As our understanding deepens, lysosomes are increasingly recognized not merely as terminal degradative compartments, but as highly responsive regulatory hubs whose dysfunction contributes directly to the initiation and progression of oxidative stress-related diseases.

### 2.2. Iron Metabolism

**Figure 2:**
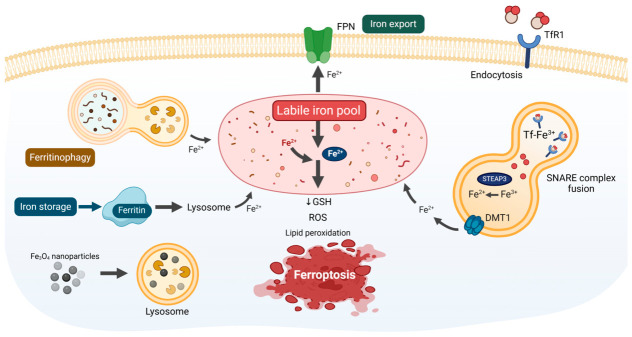
Lysosomal iron metabolism and ferroptosis regulation. This figure illustrates that transferrin-bound Fe^3+^ enters cells via TfR1 and is reduced to Fe^2+^ by STEAP3, which is subsequently transported into cytosol via DMT1 and incorporated into LIP. Iron storage in ferritin and its degradation via ferritinophagy further release Fe^2+^ from lysosomes. Fe_3_O_4_ nanoparticles processed in lysosomes also provide an additional iron source. FPN exports Fe^2+^ to the extracellular space. Elevated Fe^2+^ promotes ROS generation, GSH depletion, and lipid peroxidation, ultimately triggering ferroptosis. DMT1, divalent metal transporter 1; FPN, ferroportin; GSH, glutathione; LIP, labile iron pool; ROS, reactive oxygen species; STEAP3, six-transmembrane epithelial antigen of the prostate 3; TFR1, transferrin receptor 1. Created in BioRender. Luo, T. (2026) https://BioRender.com/kn2t93w (accessed on 1 July 2026).

Iron overload is a defining biochemical feature of ferroptosis. As an essential cofactor for DNA replication, mitochondrial respiration, and antioxidant enzyme activity, iron must be maintained within a narrow physiological range to sustain cellular viability [41]. This balance is governed by regulated pathways controlling iron uptake, storage, and export, as illustrated in Figure 2. Disruption of these mechanisms, either through dysregulated endogenous metabolism or excess exogenous iron, leads to pathological iron accumulation that accelerates ferroptosis [1].

In vertebrate cells, iron in the circulatory system is transported by Tf, which binds Fe^3+^ and delivers it to target tissues through TfR1-mediated endocytosis [42]. The Tf-TfR1 complex traffics through the endosomal system and subsequently fuses with lysosomes via the soluble N-ethylmaleimide-sensitive factor attachment protein receptor (SNARE) complex to form endolysosomes [43]. Within this acidic compartment, ferrireductases convert Fe^3+^ into Fe^2+^, enabling its release into the cytosolic labile iron pool (LIP).

STEAP3, localized mainly to endosomal/endolysosomal membranes, is the principal ferrireductase responsible for the reduction of Fe^3+^ to Fe^2+^, which is the bioactive form of cellular transport, membrane translocation, and incorporation into ferritin [1]. The biochemical activity of STEAP3 suggests that excessive ferrireductase function could expand the LIP, which in turn could destabilize iron homeostasis and sensitize cells to ferroptosis. STEAP3 activity itself is modulated by the luminal pH, which is maintained by vacuolar-type ATPase (V-ATPase)-driven proton influx [44].

Fe^2+^ released into the cytosol enters the LIP, where it is transiently buffered, chelated mainly by the free thiol group of reduced GSH [45]. The consequences of GSH consumption during this buffering process, and its potential contribution to GSH depletion-driven ferroptosis, remain insufficiently explored. Excess intracellular iron is subsequently stored in ferritin or hemosiderin, making the precise regulation of ferritin synthesis and degradation essential for maintaining iron homeostasis [46]. Ferritinophagy, discussed in detail in later sections, is a critical determinant of this balance.

Iron export is mediated by ferroportin 1 (FPN), the sole known mammalian iron efflux transporter, which releases Fe^2+^ to the extracellular milieu where it is oxidized to Fe^3+^ by ferroxidases such as ceruloplasmin (CP) and hephaestin (HEPH) [47]. Dysregulation of FPN-dependent iron efflux has been implicated in neurodegeneration. Conditional deletion of FPN in mice results in hippocampal iron retention, ferroptosis-associated molecular signatures, and significant cognitive decline [48]. Conversely, restoring FPN expression alleviates ferroptosis-related polyunsaturation and improves behavioral outcomes in Alzheimer’s disease (AD) models [48].

In addition to endogenous dysregulation, exogenous iron sources can contribute to ferroptosis susceptibility. Fe_3_O_4_ magnetic nanoparticles (Fe_3_O_4_-NPs), widely used in targeted drug delivery and biomedical imaging, are efficiently endocytosed and predominantly accumulate in lysosomes [49,50,51]. Their subsequent degradation releases iron into the LIP, indicating that lysosomes serve as a central processing hub for nanoparticle-derived iron [52,53]. Excessive iron liberated through this mechanism has been shown to disturb intracellular iron equilibrium and potentially trigger ferroptosis [54,55].

### 2.3. Lipid Peroxidation

Lipid peroxidation is a defining biochemical hallmark of ferroptosis, characterized by the iron-dependent oxidation of PUFA-containing phospholipids and the accumulation of membrane-disruptive peroxidized lipid species [56]. This process is initiated when radicals attack phospholipid chains, leading to structural destabilization and progressive membrane injury. The Fenton reaction is a major source of ROS, in which intracellular Fe^2+^ donates electrons to peroxides to generate hydroxyl radicals, thereby amplifying oxidative stress and accelerating lipid peroxidation [57]. Accumulation of peroxidized phospholipids has been observed not only in the endoplasmic reticulum membrane but subsequently in the plasma membrane, signifying a progressive propagation pattern that ultimately compromises membrane integrity [57].

Since ferroptosis is driven by oxidative stress, identifying the key antioxidant defense mechanisms involved in redox balance is essential for elucidating its underlying pathways. Failure of antioxidant surveillance is a prerequisite for ferroptosis lipid peroxidation. Under GPX4-deficient conditions, cells fail to neutralize lipid peroxides, allowing oxidized phospholipids to accumulate to lethal levels and drive ferroptosis [56]. As the key lipid repair enzyme, GPX4 catalyzes the reduction of PLOOH using GSH as an essential cofactor [58]. Intracellular GSH availability is dependent on cystine uptake, which is mediated by the cystine/glutamate transporter (System Xc−), a transmembrane transport protein located on the cell membrane that exchanges extracellular cystine for intracellular glutamate. Its functional activity is typically positively correlated with the expression level of the solute carrier family 7 member 11 (SLC7A11) light chain subunit, and transcriptional repression of SLC7A11 has been shown to sensitize cells to ferroptosis. Notably, unlike p53-mediated regulation, activating transcription factor 3 promotes erastin-induced ferroptosis by binding to the SLC7A11 promoter and suppressing its expression [59]. Collectively, the System Xc−GSH-GPX4 axis represents a significant determinant of intracellular redox homeostasis and remains a critical therapeutic target for modulating ferroptotic susceptibility.

The enzymatic machinery governing PUFA enrichment and oxidation in phospholipids forms another essential layer of ferroptotic regulation. Among PUFAs, arachidonic acid (AA) and adrenic acid (AdA) exhibit the highest susceptibility to peroxidation during ferroptosis [1,56]. Acyl-CoA Synthetase Long Chain Family Member 4 (ACSL4) catalyzes the esterification of free PUFAs, thereby determining substrate availability for incorporation into membrane lipids [23,60,61]. Subsequently, lysophosphatidylcholine acyltransferase 3 facilitates the incorporation of these activated PUFAs into phospholipids, shaping the PUFA-phospholipid landscape of cellular membranes [22]. Lipoxygenases then catalyze the selective peroxidation of PUFA-phospholipids (PUFA-PLs), generating various lipid hydroperoxides that function as cytotoxic executors of ferroptosis [5,28]. Among these oxidized products, PLOOH possess the highest membrane-disrupting potential and directly precipitates ferroptosis. Ferroptosis sensitivity is strongly influenced by cellular lipid composition. Elevated PUFA availability enhances lipid peroxidation and accelerates ferroptosis execution. Dietary or pharmacological potentiation of ω-3 PUFAs, particularly eicosapentaenoic acid and docosahexaenoic acid, has been shown to markedly increase ferroptosis susceptibility, while epidemiological evidence suggests cancer-preventive implications associated with enhanced ferroptosis-mediated elimination of pre-malignant cells [62]. Recent findings also highlight immune-mediated metabolic rewiring, showing that cytotoxic T lymphocyte-derived IFN-γ upregulates ACSL4 expression and promotes tumor cell incorporation of AA into C16/C18-acylated phospholipid species, thereby facilitating immunogenic ferroptosis [63]. Furthermore, PKCβII senses emerging lipid peroxides and amplifies lipid oxidation by phosphorylating and activating ACSL4, thereby promoting ferroptosis execution [64].

**Figure 3:**
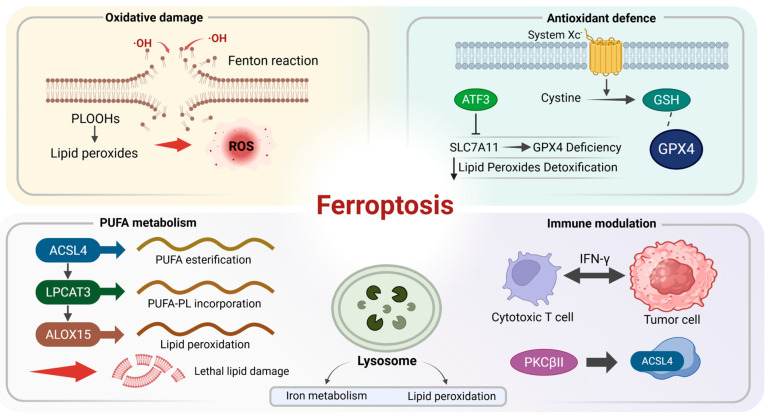
Lipid peroxidation is the central driver of ferroptosis. The upper panels depict iron-dependent oxidative damage driven by Fenton reactions and the opposing antioxidant defense mediated by the System Xc−GSH-GPX4 axis, with ATF3-dependent repression of SLC7A11 further shifting redox balance toward ferroptosis. The lower panels illustrate PUFA metabolism-mediated lipid peroxidation involving ACSL4, LPCAT3, and ALOX15, as well as immune modulation by IFN-γ and PKCβII, collectively promoting ferroptosis. ACSL4, acyl-CoA synthetase long-chain family member 4; ALOX15, arachidonate lipoxygenase 15; ATF3, activating transcription factor 3; GPX4, glutathione peroxidase 4; GSH, glutathione; IFN-γ, interferon gamma; LPCAT3, lysophosphatidylcholine acyltransferase 3; PKCβII, protein kinase C beta II; PUFA-PL, polyunsaturated fatty acid-containing phospholipids; ROS, reactive oxygen species; SLC7A11, solute carrier family 7 member 11. Created in BioRender. Luo, T. (2026) https://BioRender.com/e3jx32y (accessed on 1 July 2026).

The integrated regulatory network underlying lipid peroxidation-driven ferroptosis, including redox control by the System Xc^−^–GSH–GPX4 axis, PUFA-phospholipid remodeling, and immune-mediated metabolic modulation, is illustrated in Figure 3. Notably, the biochemical cascade of lipid peroxidation establishes a mechanistic bridge to lysosomal involvement in ferroptosis, as lysosomal iron mobilization and membrane composition cooperate to amplify oxidative lipid injury, setting the stage for subsequent sections exploring lysosomal regulatory nodes in ferroptotic pathways.

## 3. Lysosome-Mediated Iron Mobilization and Selective Autophagy in Ferroptosis

Lysosomes serve as a central metabolic hub that governs ferroptosis by coordinating iron trafficking, autophagic degradation, and redox balance. Key lysosomal proteins determine the size and reactivity of the labile iron pool, thereby shaping cellular sensitivity to Fenton-driven lipid peroxidation. Through these pathways, lysosomes exert decisive control over ferroptotic vulnerability.

### 3.1. Lysosomal Membrane Proteins in Ferroptosis

#### 3.1.1. TfR1

TfR1 serves as a pivotal regulator of cellular iron acquisition and thereby critically influences ferroptosis susceptibility through regulation of intracellular iron availability. By selectively binding Tf at the plasma membrane, TfR1 initiates clathrin-mediated endocytosis, delivering iron into endosomal compartments where Fe^3+^ is released under the acidic conditions and subsequently made available to the cytosolic labile iron pool. Given the essential role of redox-active iron in driving lipid peroxidation via Fenton reaction, alterations in TfR1 expression or trafficking profoundly impact ferroptotic vulnerability.

At the post-transcriptional level, TfR1 expression is tightly governed by the iron-responsive element/iron regulatory protein (IRE/IRP) system [65,66]. Under conditions of iron deficiency or oxidative stress, IRPs bind to IREs in the untranslated regions of TFR1 mRNA, thereby stabilizing the transcript and enhancing TfR1 expression [65]. This promotes iron uptake and expands the cytosolic LIP, thereby predisposing cells to ferroptosis [65]. Thus, the IRP/IRE system functions as a rapid, transcription-independent rheostat that links cellular iron demand to ferroptotic sensitivity.

**Figure 4:**
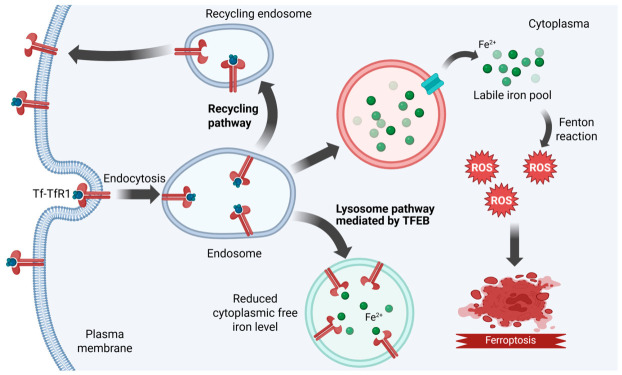
TfR1 trafficking regulates ferroptosis. This figure illustrates how TfR1-mediated iron uptake and post-endocytic trafficking regulate ferroptosis. Transferrin-bound Fe^3+^ is internalized via clathrin-mediated endocytosis, and sorting of TfR1 toward recycling or lysosomal pathways determines cytosolic iron availability. Increased labile iron drives Fenton reactions, ROS generation, and lipid peroxidation leading to ferroptosis, whereas TFEB-mediated lysosomal targeting of TfR1 promotes iron sequestration and suppresses ferroptosis. ROS, reactive oxygen species; TFEB, transcription factor EB; Tf, transferrin; TfR1, transferrin receptor 1. Created in BioRender. Luo, T. (2026) https://BioRender.com/99aeikr (accessed on 1 July 2026).

Notably, the ferroptotic outcome of TfR1-mediated iron uptake is further shaped by post-endocytic trafficking decisions that determine the intracellular fate of internalized iron, which is illustrated in Figure 4. Following clathrin-mediated endocytosis, TfR1 can be efficiently recycled back to the plasma membrane or redirected to lysosome-associated compartments, thereby influencing whether iron is released into the cytosol or retained within a buffered reservoir.

Recent studies have identified TFEB, a master regulator of lysosomal biogenesis and trafficking, as a protective modulator of TfR1-dependent iron homeostasis [67]. TFEB overexpression enhances TfR1 biosynthesis while simultaneously facilitating its trafficking to lysosomes [67]. This process promotes dynamic iron buffering capacity within lysosomes and maintains low levels of cytosolic labile iron, thereby restricting iron availability for cytosolic Fenton reactions and lipid peroxidation.

TFEB-mediated lysosomal iron sequestration confers robust resistance to ferroptotic stress by functionally uncoupling iron uptake from ferroptotic execution. Importantly, this protective effect is achieved not by suppressing TfR1 expression but by spatially compartmentalizing iron within lysosomes. Collectively, the TFEB-TfR1–lysosome axis establishes lysosomal iron handling as a critical regulatory layer in ferroptosis and a promising therapeutic target for modulating iron-dependent cell death.

#### 3.1.2. V-ATPase

V-ATPase is an ATP-driven proton pump embedded in lysosomal membranes and is indispensable for the establishment and maintenance of lysosomal acidity (pH 4.5–5.0) [68]. This acidic microenvironment constitutes a prerequisite for lysosome-dependent iron mobilization, as it supports ferritin degradation and facilitates iron release into the cytosol [12,69]. Through lysosomal ferritin catabolism, iron is liberated, contributing to LIP expansion and providing a catalytic basis for lipid peroxidation, thereby enhancing cellular susceptibility to ferroptosis.

In addition to its canonical role in lysosomal acidification, V-ATPase functions as a context-dependent regulator of ferritinophagy. Under prolonged glucose starvation, transcription factor 25 (TCF25) directly interacts with the V-ATPase V1 subunit A, thereby promoting lysosomal acidification [70]. This metabolic stress-induced activation of the V-ATPase selectively accelerates ferritin degradation, resulting in increased intracellular iron availability and ROS accumulation, ultimately sensitizing cells to ferroptotic cell death [70]. These findings highlight V-ATPase as a metabolic stress-responsive modulator that links nutrient deprivation to iron-dependent oxidative damage.

Recent evidence indicates that lysosomal acidity is governed by the balance between V-ATPase-mediated proton influx and SLC7A11-dependent proton efflux. As a slow H+ leak channel, SLC7A11 counteracts excessive acidification through cystine/glutamate exchange [71]. Its inhibition induces lysosomal over-acidification, defective degradation, and heightened ferroptotic sensitivity [71]. Aberrant lysosomal acidification facilitates iron-dependent oxidative stress, whereas restoration of lysosomal pH is sufficient to suppress lipid peroxidation and ferroptotic execution [71].

**Figure 5:**
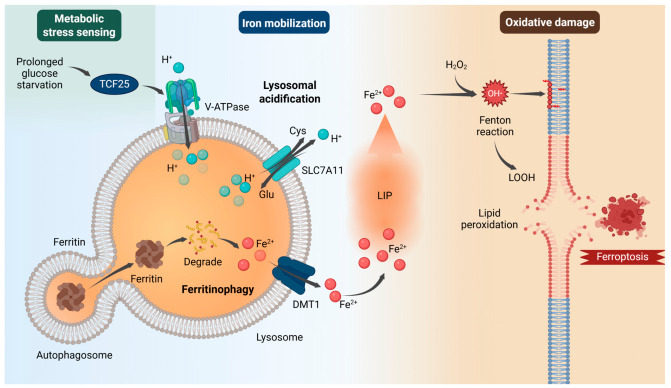
V-ATPase in ferroptosis. This figure illustrates how metabolic stress-induced lysosomal acidification regulates iron-dependent ferroptosis. The green panel depicts TCF25-mediated activation of V-ATPase during prolonged glucose starvation, enhancing lysosomal acidification and ferritin degradation. The blue panel shows lysosomal acidification-driven ferritin catabolism and Fe^2+^ export, leading to expansion of the cytosolic LIP. The orange panel illustrates LIP-derived Fe^2+^ fueling Fenton reactions, reactive oxygen species generation, and lipid peroxidation, ultimately triggering ferroptosis. DMT1, divalent metal transporter 1; LIP, labile iron pool; SLC7A11, solute carrier family 7 member 11; TCF25, transcription factor 25; V-ATPase, vacuolar-type ATPase. Created in BioRender. Luo, T. (2026) https://BioRender.com/qnc4wo5 (accessed on 1 July 2026).

V-ATPase integrates lysosomal acidification with metabolic stress sensing to govern ferritinophagy and iron release, positioning lysosomes as dynamic hubs that translate nutrient cues into ferroptotic vulnerability through iron-dependent oxidative pressure, as illustrated in Figure 5.

#### 3.1.3. STEAP3

STEAP3 is a transmembrane protein characterized by six C-terminal α-helical domains and serves as a key ferrireductase in intracellular metabolism. Functionally, STEAP3 catalyzes the reduction of Tf-derived Fe^3+^ to Fe^2+^, a prerequisite step for iron export from the endolysosomal compartment via DMT1 [72]. Notably, the redox activity of STEAP3 is strictly dependent on the acidic microenvironment of late endosomes and lysosomes, establishing lysosomal proton homeostasis as a potential master regulator of STEAP3-mediated iron handling and, consequently, cellular iron bioavailability [9].

Accumulating evidence indicates that STEAP3 can act as a potent pro-ferroptotic regulator by expanding the intracellular labile Fe^2+^ pool under specific pathological conditions. By accelerating Fe^3+^ reduction within lysosomes, STEAP3 enhances the availability of redox-active iron, thereby promoting lipid peroxidation and ferroptotic cell death. In the fatty liver transplantation model, Wu et al. (2022) demonstrated that miR-124-3p, derived from heme oxygenase-1-modified bone marrow mesenchymal stem cell-derived exosomes, suppresses STEAP3 expression in vascular endothelial cells, leading to attenuated ferroptosis and alleviation of ischemia–reperfusion injury [73]. Similarly, Deng et al. elucidated that chronic cadmium exposure induces STEAP3-dependent lysosomal iron overload, triggering glutathione depletion, excessive oxidative stress, and ferroptotic renal injury [74]. These studies collectively support a model in which STEAP3-mediated reduction of Fe^3+^ fuels ferroptotic execution by amplifying intracellular Fe^2+^ availability in stress-exposed tissues.

Paradoxically, emerging evidence indicates that STEAP3 may inhibit ferroptosis in specific oncogenic contexts, underscoring its functional duality. Intriguingly, STEAP3 inversely correlates with p53, a master regulator of ferroptosis known to repress SLC7A11 (xCT). Han et al. demonstrated in ovarian cancer models that STEAP3 knockdown activates the p53/SLC7A11 axis, resulting in enhanced ferroptotic sensitivity and impaired tumor cell survival [75]. Reinforcing this, Ye et al. revealed that genetic depletion of STEAP3 in renal cell carcinoma potentiates p53/xCT-mediated ferroptosis, thereby suppressing tumor progression [76]. These findings suggest that, in tumor cells with intact p53 signaling, STEAP3 may function as a ferroptosis-buffering factor rather than a pro-ferroptotic driver.

**Figure 6:**
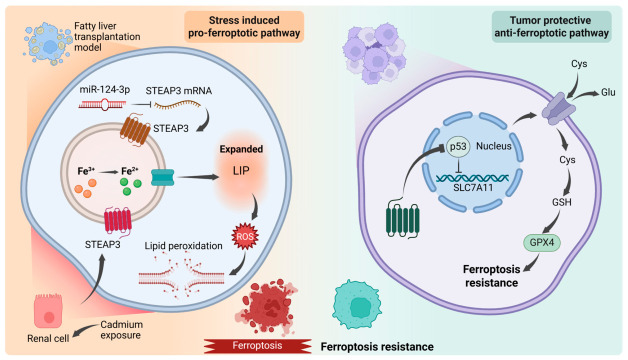
Dual roles of STEAP3 in regulating ferroptosis. This figure illustrates the bidirectional regulatory effects of STEAP3 on ferroptosis under distinct pathological conditions. The left panel depicts stress-induced pro-ferroptotic signaling, in which STEAP3 promotes lysosomal Fe^3+^ reduction and Fe^2+^ mobilization, expanding the LIP and driving ROS accumulation, lipid peroxidation, and ferroptosis. The right panel shows tumor-protective signaling, where STEAP3 modulates the p53/SLC7A11 axis, enhances glutathione synthesis and GPX4 activity, and suppresses ferroptosis. GPX4, glutathione peroxidase 4; GSH, glutathione; LIP, labile iron pool; miR-124-3p, microRNA-124-3p; ROS, reactive oxygen species; SLC7A11, solute carrier family 7 member 11; STEAP3, six-transmembrane epithelial antigen of the prostate 3. Created in BioRender. Luo, T. (2026) https://BioRender.com/n5nujsf (accessed on 1 July 2026).

Collectively, these observations position p53 signaling as a central molecular switch governing the bidirectional role of STEAP3 in ferroptosis, as summarized in Figure 6. Under conditions favoring rapid Fe^2+^ mobilization into the cytosol, STEAP3 amplifies ferroptotic vulnerability; conversely, when coupled to p53-dependent metabolic reprogramming or lysosomal iron sequestration, STEAP3 activity may attenuate ferroptotic execution.

Taken together, these observations underscore STEAP3′s context-dependent regulatory effects on ferroptosis, whose functional outcome is shaped by disease-specific microenvironments, lysosomal acidification status, and tumor suppressor signaling networks. Thus, meticulous elucidation of its disease-specific molecular circuitry is necessary for targeted therapeutic development.

#### 3.1.4. DMT1

DMT1 (also known as solute carrier family 11 member 2, SLC11A2) is a phylogenetically conserved transmembrane iron transporter composed of 12 α-helical membrane-spanning domains that mediate cellular Fe^2+^ acquisition and intracellular trafficking. It has four alternatively spliced isoforms (A-I, A-II, B-I, and B-II), among which DMT1B-I is predominantly localized to lysosomal membranes in reticuloendothelial cells, where it mediates proton-coupled iron efflux from the endolysosomal lumen into the cytosol [77]. Through this lysosome-restricted activity, DMT1 acts as a critical conduit linking lysosomal iron mobilization to the cytosolic labile iron pool, thereby exerting a decisive influence on ferroptotic sensitivity.

At the transcriptional level, DMT1 expression is dynamically regulated by diverse pathological cues, thereby modulating ferroptosis in a context-dependent manner. In atherosclerotic models, Mai-Ji-Tong (MJT) granules suppress DMT1 transcription by activating STAT6 phosphorylation, leading to attenuation of ferroptosis and disease progression [78]. Moreover, hypoxia-inducible factor-2α directly binds to the DMT1 promoter to activate its transcription in response to iron deficiency, thereby augmenting intracellular iron availability [79]. In parallel, pro-inflammatory cytokines, such as TNF-α and IL-6, upregulate DMT1 expression in neurons, astrocytes, and microglia, promoting iron accumulation and oxidative damage [80]. These transcriptional programs highlight DMT1 as an iron transporter that integrates hypoxic and inflammatory signals to regulate lysosomal iron flux and ferroptotic vulnerability.

DMT1 abundance is regulated not only at the transcriptional level but also through post-translational protein quality control mechanisms that govern its stability and turnover. In intracerebral hemorrhage models, the E3 ubiquitin ligase Nedd4 mediates site-specific ubiquitination of DMT1 at lysine residues K6, K69, and K277, promoting DMT1 degradation via the proteasome pathway, exerting its neuroprotective effect [81]. Disruption of this Nedd4-dependent ubiquitination pathway stabilizes DMT1 protein levels, augments lysosome-to-cytosol iron efflux, and exacerbates ferroptosis [81]. This regulatory axis underscores the importance of protein turnover in fine-tuning DMT1-mediated iron mobilization and identifies DMT1 protein stability as a modifiable determinant of ferroptosis.

In addition to transcriptional and post-translational regulation, ferroptotic susceptibility is critically influenced by the structural integrity and functional competence of lysosomal DMT1. Under glucose starvation, lysosomal dysfunction induces the fragmentation and degradation of DMT1 directly at the lysosomal membrane, resulting in a profound loss of its iron export capacity [82]. As DMT1 represents the primary pathway for Fe^2+^ efflux from lysosomes, its functional impairment blocks iron release and leads to pathological iron accumulation within lysosomes rather than increased cytosolic iron availability. This lysosomal iron overload promotes localized redox stress and lipid peroxidation, ultimately triggering ferroptosis. Consistently, pharmacological inhibition of DMT1 or V-ATPase recapitulates lysosomal iron accumulation and ferroptosis, whereas iron chelation effectively reverses these effects [82]. These findings establish DMT1 fragmentation as a loss-of-function event that links lysosomal dysfunction to ferroptosis by sequestering iron within lysosomes.

At a finer regulatory level, non-coding RNAs provide an additional layer of control over DMT1-mediated iron transport. In acute myocardial infarction (AMI) models, pathological upregulation of DMT1 is alleviated by exosomal miR-23a-3p derived from human umbilical cord blood mesenchymal stem cell (HUCB-MSC), which suppresses DMT1 expression and attenuates cardiomyocyte ferroptosis [83]. This miRNA-dependent modulation highlights the therapeutic potential of precisely targeting lysosome-associated iron transport without broadly perturbing systemic iron metabolism.

**Figure 7:**
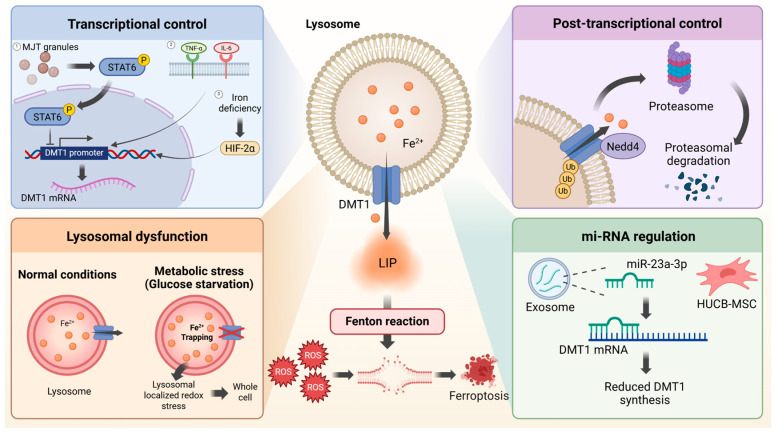
DMT1 regulation in ferroptosis. This figure illustrates the regulatory network controlling DMT1-dependent iron transport and ferroptotic sensitivity. The upper left panel depicts transcriptional regulation of DMT1 by MJT granules, hypoxia, and inflammatory cytokines via STAT6 and HIF-2α signaling, influencing lysosomal iron efflux and LIP expansion. The upper right panel shows post-translational regulation, where Nedd4-mediated ubiquitination promotes DMT1 degradation and restricts iron export. The lower left panel illustrates lysosomal dysfunction-induced DMT1 fragmentation that causes intralysosomal iron accumulation and oxidative damage. The lower right panel depicts miRNA-mediated regulation, in which HUCB-MSC-derived exosomal miR-23a-3p suppresses DMT1 expression and attenuates ferroptosis. DMT1, divalent metal transporter 1; HIF-2α, hypoxia-inducible factor 2α; HUCB-MSC, human umbilical cord blood mesenchymal stem cell; LIP, labile iron pool; MJT, Mai-Ji-Tong; ROS, reactive oxygen species. Created in BioRender. Luo, T. (2026) https://BioRender.com/ysm9znv (accessed on 1 July 2026).

Collectively, these multi-layered regulatory mechanisms converge on DMT1 to control lysosome-derived iron mobilization, thereby dictating cellular susceptibility to ferroptosis across diverse pathological contexts. By integrating transcriptional control, protein stability, lysosomal structural integrity, and non-coding RNA-mediated fine-tuning, DMT1 emerges as a central lysosomal iron gatekeeper and a promising therapeutic target in ferroptosis-related diseases, as summarized in Figure 7.

#### 3.1.5. LAMPs

As integral lysosomal membrane proteins, lysosome-associated membrane proteins (LAMPs) share a conserved structure characterized by a large luminal domain, a single transmembrane helix, and a short cytosolic tail. The luminal domains of LAMPs are extensively glycosylated, forming a protective glycocalyx that shields the lysosomal membrane and cytosolic components from autodigestion by luminal hydrolases. Through this structural specialization, LAMPs play a fundamental role in preserving lysosomal membrane integrity and sustaining lysosome-dependent catabolic processes [31].

LAMPs may influence ferroptotic susceptibility by protecting lysosomal membranes against oxidative injury. Their highly glycosylated luminal domains form a protective glycocalyxt. Therefore, alterations in LAMP expression or function may increase susceptibility to oxidative damage and subsequent LMP [15,31]. The downstream consequences of LMP in ferroptosis are discussed in detail in Section 4.1.

LAMPs also contribute to ferroptosis through the regulation of iron metabolism via autophagic pathways. TFEB-dependent lysosomal expansion, accompanied by increased LAMP expression, may enhance ferritin degradation and iron mobilization, thereby increasing ferroptotic susceptibility, as discussed in detail in Section 3.2.1.

**Figure 8:**
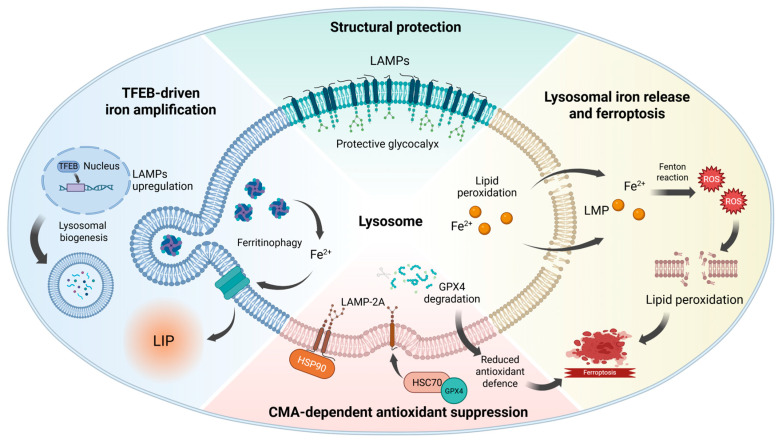
LAMPs in ferroptosis. This figure illustrates how LAMPs regulate ferroptosis through structural protection, iron mobilization, and antioxidant control. The upper central panel shows the protective glycocalyx formed by glycosylated LAMPs that maintains lysosomal membrane integrity. The left panel depicts TFEB-driven lysosomal biogenesis and ferritinophagy that expand the LIP. The right panel illustrates lipid peroxidation-induced lysosomal membrane permeabilization, leading to Fe^2+^ release, Fenton reactions, and ferroptosis. The lower panel shows CMA-mediated GPX4 degradation via LAMP-2A-dependent HSC70 import, with HSP90 stabilizing LAMP-2A to sustain CMA activity. CMA, chaperone-mediated autophagy; GPX4, glutathione peroxidase 4; HSC70, heat shock cognate 70; HSP90, heat shock protein 90; LAMP, lysosome-associated membrane protein; LMP, lysosomal membrane permeabilization; LIP, labile iron pool; ROS, reactive oxygen species; TFEB, transcription factor EB. Created in BioRender. Luo, T. (2026) https://BioRender.com/warkp19 (accessed on 1 July 2026).

LAMPs play a particularly prominent role by coordinating multiple autophagy pathways, including macroautophagy, CMA, and nucleic acid-selective autophagy, and the mechanistic involvement of individual LAMP isoforms is detailed in Table 1 [84,85]. LAMP-2 is essential for the fusion of autophagosomes and phagosomes with lysosomes, a prerequisite for the formation of autolysosomes and phagolysosomes [31,84,86]. In CMA, the cytosolic tail of LAMP-2A functions as a receptor for substrate translocation, and its expression level directly correlates with CMA activity [85]. Notably, LAMP-2A mediates the lysosomal import of the HSC70-GPX4 complex, facilitating GPX4 degradation and thereby removing a ferroptosis suppressor [18,87]. This mechanism establishes a direct molecular link between lysosomal CMA and ferroptosis. Furthermore, HSP90 stabilizes LAMP-2A through direct interaction, thereby sustaining CMA activity [87]. The integrated roles of LAMPs in preserving lysosomal membrane integrity, regulating ferritinophagy-mediated iron release, and mediating GPX4 degradation via CMA are illustrated in Figure 8.

### 3.2. Lysosome-Dependent Autophagy in Ferroptosis

Autophagy is an evolutionarily conserved lysosome-dependent mechanism for the degradation of cellular components and maintenance of metabolic homeostasis. Autophagy not only coexists with apoptosis but also interacts synergistically with ferroptosis [88]. Accumulating evidence indicates that ferroptosis is an autophagy-dependent form of cell death. Mechanistic studies have elucidated the essential regulatory role of autophagy in ferroptosis execution. Experimental models treated with classical ferroptosis inducers (e.g., erastin and RSL3) consistently exhibit enhanced autophagic flux, a phenomenon confirmed across multiple cell types [88].

Emerging evidence highlights three essential mechanisms by which autophagy influences ferroptosis. First, autophagy promotes ferroptosis by degrading regulatory factors that suppress ferroptosis [56]. Second, it facilitates the generation of ferroptosis-associated ROS and lipid peroxides [89,90]. Notably, this process is independent of GSH depletion, suggesting that inhibiting autophagy effectively prevents the lethal accumulation of lipid peroxides [89]. Third, autophagy promotes ferroptosis by mediating ferritinophagy and iron release [13]. Among these mechanisms, ferritinophagy provides a direct link between lysosomal degradation and iron-dependent oxidative damage. Nevertheless, the role of autophagy in ferroptosis is context-dependent. While autophagy generally serves as a self-preservation mechanism that mitigates cellular damage under stress [91,92], its hyperactivation or dysregulation, particularly under conditions of redox imbalance and excessive ROS accumulation, can contribute to autophagic cell death [93].

#### 3.2.1. Ferritinophagy

Accumulating evidence indicates that erastin-induced ferroptosis is tightly regulated by autophagy-mediated iron homeostasis disruption. Mechanistically, erastin-induced autophagy expands the intracellular labile iron pool through lysosomal degradation of ferritin and transcriptional upregulation of TfR1, thereby enhancing iron uptake [90]. This iron overload synergizes with erastin-induced ROS to amplify lipid peroxidation, expediting ferroptotic cell demise. Notably, a self-reinforcing loop emerges wherein ROS not only serve as terminal effectors of ferroptosis but also function as upstream modulators that sustain autophagic flux, establishing a feedforward mechanism that accelerates ferroptosis progression.

NCOA4 functions as a selective cargo receptor for ferritinophagy, a lysosomal degradation pathway dedicated to the turnover of iron-storage ferritin complexes [12]. Experimental evidence reveals that during ferroptotic cascades, NCOA4 and its binding partner, ferritin heavy chain (FTH), undergo targeted proteolytic degradation within the autophagy-lysosome system [89,94]. NCOA4 plays a pivotal role in modulating ferroptosis. Its depletion disrupts lysosomal trafficking and degradation of ferritin, resulting in decreased intracellular iron availability and subsequent dysregulation of iron metabolism and homeostasis. Knockout of NCOA4 suppresses ferroptotic cell death by attenuating lipid peroxidation and ROS generation [89], concomitant with an upregulation of FTH1 expression. Conversely, NCOA4 overexpression amplifies ferroptotic susceptibility by promoting iron-dependent oxidative stress [13].

Sequestosome 1 (SQSTM1) functions as a multifunctional scaffold protein that serves as a critical molecular hub integrating ferritinophagy and ferroptotic signaling. Meanwhile, ubiquitin-specific peptidase 8 (USP8), a key deubiquitinating enzyme, governs the stability and functional dynamics of SQSTM1 by precisely modulating its ubiquitination status. Erastin-induced ubiquitination of SQSTM1 at lysine 420 (K420) enhances autophagic flux, promoting the degradation of ferritin light chain (FTL) and NCOA4. Mechanistically, SQSTM1 functions as a molecular scaffold that anchors NCOA4 to microtubule-associated protein 1 light chain 3, a key marker of autophagosome biogenesis, thereby amplifying the ferritinophagy cascade and facilitating iron-dependent proteolysis. Activation of the advanced glycosylation end-product-specific receptor, through its functional interplay with recombinant SQSTM1 protein, induces transcriptional upregulation of ACSL4, a rate-limiting enzyme in PUFA biosynthesis. This pathway not only amplifies cellular susceptibility to autophagy-dependent ferroptosis via PUFA-mediated lipid peroxidation cascades but also modulates inflammatory signaling networks, potentially bridging iron-dependent cell death with immune microenvironment remodeling. USP8 functions as a pivotal regulator in suppressing ferritinophagy-mediated ferroptosis. Mechanistically, USP8 removes ubiquitin modifications from SQSTM1, primarily at lysine 420 (K420), thereby suppressing SQSTM1-mediated ferritinophagy. Pharmacological inhibition of USP8 significantly accelerates erastin-induced ferritin degradation via the ferritinophagy pathway in HepG2 cells, consequently heightening cellular susceptibility to ferroptosis [95]. Notably, exogenous overexpression of SQSTM1/p62 exacerbates autophagy-dependent ferroptotic cell death, highlighting the potential of SQSTM1-targeted suppression as a novel therapeutic strategy for acute pancreatitis [95].

Moreover, emerging evidence indicates that lysosomal activation is a key regulator of ferroptosis via ferritinophagy-mediated iron release. Mechanistically, nuclear translocation of the lysosomal master regulator TFEB orchestrates the transcriptional upregulation of lysosomal hydrolases (e.g., cathepsins) and autophagic machinery components. In hepatocellular carcinoma models, quercetin promotes TFEB nuclear translocation and lysosomal biogenesis, thereby enhancing ferritin degradation, iron mobilization, and ferroptotic susceptibility [96,97].

#### 3.2.2. CMA-Mediated Regulation

CMA is a selective form of autophagy characterized by receptor-mediated substrate recognition and translocation across the lysosomal membrane. Typically induced under stress conditions, CMA plays a crucial role in the proteolytic clearance of key ferroptosis-associated proteins through its substrate-specific degradation machinery.

ACSL4 serves as both a ferroptosis biomarker and a key driver of its execution. Upregulation of ACSL4 enhances ferroptotic susceptibility by enriching phospholipids with oxidation-prone PUFAs, thereby fostering a lipid microenvironment conducive to iron-catalyzed peroxidation cascades [1,23]. Its degradation is tightly controlled by the chaperone HSC-70, which mediates its lysosomal recognition and subsequent proteolysis [98]. Impairment of autophagic-lysosomal flux disrupts ACSL4 homeostasis, leading to its pathological accumulation. This aberrant increase amplifies the generation of cytotoxic lipid peroxidation substrates, thereby exacerbating ferroptotic cell death in retinal pigment epithelium (RPE) under metabolic stress [99].

Glia maturation factor-β (GMF-β), a neuroinflammatory mediator significantly upregulated in the vitreous humor during early diabetic retinopathy (DR), has emerged as a key regulator of DR pathogenesis. Mechanistically, GMF-β promotes retinal ferroptosis by disrupting chaperone-mediated autophagy, thereby impairing lysosomal degradation of ACSL4 and leading to its pathological accumulation [98]. This discovery highlights a previously unrecognized interplay between protein quality control mechanisms and iron-dependent cell death in DR progression, providing a molecular framework for targeting the GMF-β/ACSL4 axis in early therapeutic interventions.

CMA indirectly modulates intracellular GPX4 levels through LAMP-2A, a lysosomal membrane-bound substrate receptor. Studies reveal that erastin promotes CMA by elevating LAMP-2A expression, leading to GPX4 degradation and ferroptosis activation [18]. This mechanistic insight positions LAMP-2A-driven CMA as a promising therapeutic avenue in oncology.

#### 3.2.3. Clockophagy

Clockophagy is a novel selective autophagy process involving the autophagic degradation of the core circadian clock protein aryl hydrocarbon receptor nuclear translocator-like protein 1 (ARNTL). Mechanistically, ARNTL represses Egl-9 homolog 2 transcription through direct promoter binding, thereby maintaining hypoxia-inducible factor 1α expression, a transcription factor that promotes cell survival under hypoxic conditions [100]. This discovery unveils a new pathway in which autophagic removal of ARNTL facilitates ferroptosis. Depletion of ARNTL via clockophagy disrupts this regulatory axis, enabling Egl-9 homolog 2-mediated lipid peroxidation to drive ferroptosis [101]. Crucially, during ferroptosis induced by type 2 ferroptosis inducers, SQSTM1/p62 functions as the lysosomal degradation receptor that targets ARNTL for lysosomal degradation [101,102].

### 3.3. Mitochondria–Lysosome Crosstalk in Ferroptosis

The role of lysosomes in ferroptosis is not limited to their intrinsic functions in iron mobilization and autophagic degradation. Lysosomes also interact with mitochondria through physical contact, interorganelle material exchange, and mitophagy, thereby reshaping the intracellular distribution of iron, ROS, and lipid peroxidation substrates. In addition, the spatial organization of mitochondrial membranes may influence the assembly and amplification of these signals.

Mitochondria form direct and dynamic contact sites with lysosomes, and recent evidence indicates that these contacts participate in iron redistribution between the two organelles [103]. The enzyme 3-hydroxybutyrate dehydrogenase 2 (BDH2) localizes at mitochondria–lysosome contact sites and generates 2,5-dihydroxybenzoic acid, a siderophore-like molecule that facilitates Fe^2+^ transfer from lysosomes to mitochondria [104]. BDH2 depletion impairs this process, resulting in lysosomal Fe^2+^ retention and increased ferroptotic susceptibility. These findings indicate that ferroptosis depends not only on total cellular iron content but also on its subcellular distribution. In contrast, in perfluorooctane sulfonate-exposed hepatocytes, autophagy-dependent lysosomal iron accumulation is followed by iron transfer to mitochondria through a proposed interaction between the lysosomal channel transient receptor potential mucolipin 1 (TRPML1) and the mitochondrial outer membrane protein voltage-dependent anion-selective channels 3 (VDAC3), leading to mitochondrial iron overload and ferroptosis [105]. Thus, lysosome-to-mitochondria iron transfer appears to exert context-dependent effects.

Mitochondria are major sites of iron utilization and may also temporarily buffer intracellular iron. However, when mitochondria are degraded by lysosomes through mitophagy, mitochondrial iron can be remobilized. Mitophagy therefore has a dual role in ferroptosis. On the one hand, the removal of damaged mitochondria reduces mitochondrial ROS production and protects cells against ferroptosis [106]. On the other hand, lysosomal degradation of iron-containing mitochondria can release iron and provide additional substrates for Fenton reactions [107]. Mitophagy-associated lipid mobilization may also increase the availability of free fatty acids for lipid peroxidation [108]. Consequently, the effect of mitophagy on ferroptosis depends on the balance between the clearance of ROS-producing mitochondria and the release of pro-oxidant iron and lipid substrates.

The effects of mitochondria–lysosome crosstalk may also depend on the spatial organization of mitochondrial membranes. Mitochondrial raft-like microdomains, enriched in specific lipids such as cardiolipin, can serve as platforms for the recruitment of proteins involved in cell death, mitochondrial fission, and autophagy [109,110,111,112]. For example, these domains facilitate the organization of mitochondrial fission 1 protein (FIS1) and dynamin-related protein 1 (DRP1) and may thereby influence mitochondrial dynamics and subsequent lysosomal degradation. Although most evidence has been obtained from apoptosis and general autophagy models, VDAC1 oligomerization has been shown to promote mitochondrial ROS production, lipid peroxidation, and ferroptosis, suggesting that mitochondrial membrane organization may also influence ferroptotic susceptibility [113].

Overall, mitochondria–lysosome crosstalk regulates ferroptosis through iron redistribution, mitophagy, and membrane-associated signaling. These processes may either remove ROS-producing mitochondria or release iron and lipid substrates that promote oxidative damage. However, whether raft-like microdomains directly regulate mitochondria–lysosome contacts or interorganelle iron transfer during ferroptosis remains unclear.

## 4. The Involvement of Lysosome in Lipid Peroxidation in Ferroptosis

### 4.1. Lysosomes as Initiation Sites of Lipid Peroxidation

Accumulating evidence indicates that lysosomes function as preferential initiation sites of lipid peroxidation during ferroptosis. This property can be mechanistically attributed to three converging features: the intrinsic oxidizability of the lysosomal membrane, a pro-oxidant intraluminal microenvironment, and a relative insufficiency of local antioxidant defenses.

At the structural level, the lysosomal membrane is highly susceptible to oxidative damage. It is enriched in polyunsaturated fatty acid (PUFA)-containing phospholipids, which constitute the primary execution substrates of ferroptosis and are highly prone to peroxidation. Consistent with this vulnerability, recent studies have indicated that membrane lipid oxidation is preferentially initiated at the lysosomal membrane, accompanied by increased membrane permeability. Lysosomal lipid peroxidation promotes LMP, which facilitates ferroptotic cell death [15]. Notably, the ferroptosis inducer RSL3 has been shown to trigger early lipid oxidation within lysosomes, which later spreads to other cellular organelles, such as the endoplasmic reticulum, further supporting the notion that lysosomal membranes represent a primary subcellular source of lipid peroxidation reactions during ferroptosis [114].

This vulnerability is further reinforced by the distinctive intraluminal conditions of lysosomes. Lysosomes maintain a highly acidic pH and are enriched in redox-active Fe^2+^, creating sustained conditions for ROS generation [15]. This confined, acidic, and iron-rich milieu constitutes an ideal reaction compartment for Fenton reaction, thereby amplifying local oxidative stress and driving lipid peroxidation at the lysosomal membrane [15]. Emerging evidence further indicates that lysosomal iron is a critical determinant of ferroptosis sensitivity [114]. Accordingly, maintenance of lysosomal acidity is considered a prerequisite for efficient Fenton-driven lipid peroxidation within this organelle [14].

Importantly, the lysosomal membrane is relatively deficient in antioxidant protection compared with other cellular compartments. The Xc−GSH-GPX4 axis constitutes the core defense machinery against ferroptosis [21]. However, the SLC7A11/Xc− primarily operates at the plasma membrane to sustain cytosolic GSH levels, while GPX4 exerts its lipid peroxide-reducing activity predominantly in the cytosol, mitochondria, and nucleus [115]. In contrast, the lysosomal membrane lacks robust, locally adequate GPX4-dependent detoxification capacity. This spatial mismatch between sites of lipid peroxidation and antioxidant defense creates a localized redox vulnerability, rendering lysosomes an early amplifier of lipid peroxidation and ferroptotic signaling.

### 4.2. Iron-Driven Lipid Peroxidation Within Lysosomes

Lysosomal iron constitutes a redox-active iron pool rather than an inert storage compartment, with iron predominantly present in the ferrous (Fe^2+^) state, thereby providing an essential chemical prerequisite for the Fenton reaction [116]. Owing to the acidic luminal environment and the enrichment of redox-active iron, lysosomes function as spatially confined reaction chambers that favor the generation of hydroxyl radicals (HO·). These highly reactive species directly attack biomolecules undergoing degradation within the lysosomal lumen and, critically, initiate lipid peroxidation of the polyunsaturated fatty acid-rich lysosomal membrane [116]. The resulting oxidative membrane damage may culminate in LMP, as discussed in Section 4.1.

Notably, the consequences of lysosomal iron redox activity are highly context-dependent. In senescent cells, lysosomal alkalization impairs iron efflux and results in aberrant accumulation of free Fe^2+^ within the lysosomal lumen, rather than its redistribution to the cytoplasm [14,117]. At the acute level, this spatial sequestration of iron limits cytosolic lipid peroxidation and attenuates ferroptosis induction, conferring cellular resistance to ferroptosis inducers such as erastin [14,117,118]. However, this spatial isolation of iron does not affect its chemical reactivity. Instead, excessive intralysosomal iron amplifies Fenton reaction-driven oxidative damage within the lumen, leading to persistent accumulation of non-degradable lipofuscin [119].

Lipofuscin is composed of iron-rich, autofluorescent aggregates that further sensitize lysosomes to oxidative stress, creating a self-perpetuating cycle of iron retention and redox imbalance [120]. Collectively, these observations suggest that altered lysosomal iron handling during cellular senescence may transiently suppress ferroptotic vulnerability at the cytoplasmic level while exacerbating chronic lysosomal dysfunction. This spatial reprogramming of iron-driven lipid peroxidation highlights lysosomes as a critical subcellular determinant of ferroptosis susceptibility, particularly in aging-associated cellular states.

## 5. Lysosome-Centered Regulation of Antioxidant Systems in Ferroptosis

### 5.1. Lysosomal Regulation of the System Xc−GSH-GPX4 Axis

Lysosomes exert a critical influence on ferroptosis by modulating cellular antioxidant capacity, particularly through the System Xc−GSH-GPX4 axis. Lysosomes influence cystine availability and glutathione homeostasis, thereby shaping cellular susceptibility to lipid peroxidation-driven cell death.

System Xc−, composed of SLC7A11 and SLC3A2, is the primary transporter responsible for cystine uptake. Emerging evidence reveals a non-transcriptional mechanism linking the autophagy machinery to the direct suppression of System Xc− activity. Specifically, the core autophagy regulatory protein Beclin 1 (BECN1) directly binds to the System Xc− subunit SLC7A11 and inhibits system Xc− activity in a manner that depends on AMP-activated protein kinase (AMPK)-mediated phosphorylation of BECN1 [121,122]. This interaction leads to inhibition of cystine import and depletion of intracellular GSH.

As GPX4 activity is strictly dependent on adequate GSH supply, perturbations at the level of System Xc− effectively attenuate the antioxidant systems, thereby promoting the susceptibility to ferroptosis.

GSH also mediates redox signaling through the reversible S-glutathionylation of redox-sensitive cysteine residues. This modification can alter the activity of protein kinases and transcription factors, as demonstrated for MAPK (mitogen-activated protein kinase)/ERK (extracellular-signal-regulated kinase) kinase kinase 1 (MEKK1), inhibitor of nuclear factor κB kinase subunit β (IKKβ), and the p50 subunit of nuclear factor κB (NF-κB) [123,124,125]. Similarly, thioredoxin proteins participate in redox signaling through reversible thiol–disulfide switching. Reduced thioredoxin directly binds to and inhibits apoptosis signal-regulating kinase 1 (ASK1), whereas its oxidation promotes dissociation from ASK1 and subsequent activation of downstream c-Jun N-terminal kinase and p38 signaling [126]. Thioredoxin can also regulate the DNA-binding activity of NF-κB by controlling the redox state of critical cysteine residues [127]. Therefore, lysosome-dependent alterations in cysteine availability, GSH homeostasis, and intracellular redox balance may influence ferroptosis not only by impairing GPX4-mediated lipid peroxide detoxification but also by reshaping redox-sensitive signaling networks. Consistent with this possibility, thioredoxin reductase deficiency has been associated with impaired autophagy–lysosomal degradation, whereas thioredoxin reductase 1 (TXNRD1) inhibition can promote ferroptosis [128,129]. However, the direct mechanistic links among lysosomal regulation, thioredoxin-dependent redox signaling, and ferroptosis remain insufficiently defined.

### 5.2. Lysosome-Mediated Cysteine Supply Confers Ferroptosis Resistance in Cancer

Cancer cells have evolved a lysosome-dependent cysteine salvage pathway to circumvent ferroptosis triggered by extracellular cysteine deprivation. When canonical cystine uptake through System Xc− is compromised, cancer cells can internalize extracellular proteins enriched in cysteine, most notably albumin, and route them to lysosomes for proteolytic degradation [16]. Within lysosomes, albumin is primarily degraded by cathepsin B (CTSB), generating cystine, which is subsequently exported into the cytoplasm via the lysosomal transporter cystinosin. The released cystine is rapidly reduced to cysteine and incorporated into GSH biosynthesis, thereby restoring intracellular redox buffering capacity, inhibiting lipid peroxidation, and conferring resistance to ferroptosis under cysteine-limiting conditions [16]. Importantly, this pathway represents a lysosome-centered bypass mechanism that functionally decouples intracellular cysteine availability from extracellular cystine supply. Unlike the System Xc−GSH-GPX4 axis, which directly links plasma membrane transport to antioxidant defense, lysosomal cysteine salvage relies on proteolytic catabolism and vesicular trafficking, highlighting the lysosome as an active metabolic hub rather than a passive degradative organelle in the regulation of ferroptosis.

### 5.3. Lysosomal Stress Signaling: Mechanistic Target of Rapamycin (mTOR)-TFEB Axis in Ferroptosis Resistance

The mTOR-TFEB signaling axis constitutes a key lysosome-centered stress response pathway that modulates cellular redox homeostasis and ferroptosis susceptibility. Ferroptosis is a process tightly linked to intracellular ROS accumulation. Accordingly, adaptive mechanisms that restrain oxidative stress can profoundly influence the execution of ferroptosis.

mTOR is a master regulator of cell growth, survival, metabolism, and immune regulation [130]. TFEB, a master regulator of lysosomal biogenesis and autophagy, is a direct substrate of mTOR [19,38]. Under conditions of nutrient deprivation or cellular stress, mTOR inhibition decreases TFEB phosphorylation, thereby promoting its nuclear translocation [19,38]. Once activated, TFEB enhances the transcription of lysosome-related genes, including those encoding lysosomal enzymes, membrane proteins, and other factors involved in lysosomal function and autophagy [19,38,40].

TFEB can induce the expression of genes encoding antioxidant enzymes, including superoxide dismutase (SOD), thereby augmenting ROS-scavenging capacity and mitigating oxidative stress [99]. Through this mechanism, the lysosomal stress-mTOR-TFEB axis functions as an adaptive response that links lysosomal remodeling to redox control, with important consequences for ferroptosis regulation [99].

Studies have shown that carboxylated polystyrene nanoparticles effectively inhibit ferroptosis by reducing ROS levels, highlighting the critical role of lysosomes in this process [99]. Carboxylated polystyrene nanoparticle treatment induces lysosomal perturbation, leading to mTOR inactivation and TFEB nuclear translocation. This response enhances the expression of lysosomal proteins and antioxidant enzymes, including SOD, resulting in reduced intracellular ROS accumulation and effective suppression of ferroptosis [99]. Therefore, in the development of ferroptosis-inducing anticancer therapies, it is essential to consider strategies that minimize lysosomal stress and its consequent TFEB nuclear translocation to prevent the suppression of ferroptosis by intrinsic regulatory mechanisms.

## 6. Therapeutic Potential and Translational Limitations of Targeting Lysosomal Pathways

### 6.1. Disease-Specific Roles and Therapeutic Opportunities

Ferroptosis has been implicated in cancer and diverse forms of tissue injury, but the contribution of lysosomes is highly context-dependent [3]. By regulating iron mobilization, ferritin degradation, nutrient recycling, lysosomal membrane integrity, and antioxidant capacity, lysosomes can either restrain or facilitate ferroptosis.

Tumor cells can exploit lysosomal nutrient recycling and cysteine homeostasis to maintain antioxidant capacity and evade ferroptosis, creating a potential metabolic vulnerability. As discussed above, lysosomal degradation of extracellular cysteine-rich proteins provides cancer cells with an alternative source of cysteine, thereby sustaining GSH synthesis and ferroptosis resistance under nutrient-limited conditions [16]. Swanda et al. further demonstrated that a decrease in lysosomal cystine, rather than cytosolic cysteine depletion alone, triggers a kynurenine–aryl hydrocarbon receptor (AhR)-dependent transcriptional response that induces activating transcription factor 4 (ATF4) [131]. Blocking lysosomal cystine efflux attenuated ATF4 induction and sensitized cancer cells to ferroptosis, indicating that lysosomal cystine dynamics function as a metabolic checkpoint for adaptation to cysteine limitation [131]. Thus, lysosomes support tumor survival not only by controlling iron availability but also by maintaining cysteine-dependent antioxidant defenses.

Ferroptosis has also emerged as an important mechanism of neural-cell injury under oxidative stress. Tian et al. found that knockdown of the lysosomal protein prosaposin (PSAP) impaired lysosomal glycosphingolipid catabolism in human neurons, leading to lipofuscin formation. The resulting lipofuscin trapped iron, promoted ROS generation, and triggered neuronal ferroptosis, providing a mechanistic basis for lysosome-associated neuronal vulnerability relevant to aging and neurodegenerative disorders [132]. A distinct lysosome-associated mechanism has recently been identified in multiple system atrophy. In oligodendrocytes, pathological α-synuclein accumulation stabilized NCOA4, enhanced NCOA4-dependent FTH1 degradation, and promoted toxic iron accumulation and ferroptosis [133]. These studies, therefore, reveal two different routes to ferroptotic vulnerability in neural cells: PSAP loss promotes lipofuscin-associated iron trapping through defective glycosphingolipid catabolism, whereas pathological activation of ferritinophagy increases iron availability through FTH1 degradation.

NCOA4-mediated ferritinophagy also represents a shared pathological mechanism across distinct cardiovascular cell types. In atherosclerosis, Zheng et al. identified endothelial dipeptidyl peptidase 4 (DPP4) as an upstream regulator of ferritinophagy-driven ferroptosis. DPP4 upregulation promoted autophagy and NCOA4-dependent ferritinophagy, resulting in FTH1 destabilization, endothelial ferroptosis, and endothelial dysfunction, thereby contributing to atherosclerotic progression [134]. In heart failure, Deng et al. showed that N-acetyltransferase 10 (NAT10)-mediated N4-acetylcytidine modification increased the stability and translation of sterol O-acyltransferase 1 (SOAT1) mRNA. Increased SOAT1 expression enhanced NCOA4 acetylation and protein stability, thereby promoting NCOA4-dependent ferritinophagy, intracellular iron accumulation, lipid peroxidation, and cardiomyocyte ferroptosis. Consistently, SOAT1 knockdown alleviated pressure overload-induced cardiac remodeling and dysfunction, whereas NCOA4 overexpression weakened these protective effects [135]. These findings suggest that distinct upstream signals in endothelial cells and cardiomyocytes can converge on the NCOA4–FTH1 ferritinophagy pathway to amplify ferroptotic cardiovascular injury.

Inflammatory and ischemic signals can similarly engage NCOA4-dependent ferritinophagy. In sepsis, the interaction between stimulator of interferon genes (STING) and NCOA4 promoted ferritinophagy-associated macrophage ferroptosis and inflammatory responses, thereby exacerbating multiple-organ injury and mortality [136]. During ischemic acute kidney injury, STING interacted with NCOA4 to facilitate ferritin delivery to lysosomes and ferritinophagic degradation in renal tubular cells. This process increased iron availability and lipid peroxidation, whereas genetic deletion or pharmacological inhibition of STING reduced tubular ferroptosis, tissue injury, and renal dysfunction [137]. These studies suggest that the STING–NCOA4 axis connects inflammatory and ischemic stress with ferritinophagy-driven ferroptosis in both immune and parenchymal cells.

Collectively, these findings demonstrate that the pathological consequences of lysosome-dependent ferroptosis are determined by the specific substrates processed and the cellular context. Lysosomal cysteine recycling and nutrient sensing can support ferroptosis resistance in cancer, whereas abnormal lipid catabolism, iron trapping, and excessive ferritinophagy can promote ferroptotic injury in neural, cardiovascular, inflammatory, and ischemic diseases. These disease-specific dependencies provide a rationale for therapeutically targeting lysosomal pathways, while also indicating that the direction of intervention should be tailored to the disease and cell type. Representative evidence linking lysosome-dependent ferroptosis to different pathological conditions is summarized in Table 2.

These disease-specific mechanisms also reveal potentially actionable lysosomal vulnerabilities. Several small molecules and nanomaterials have therefore been investigated as preclinical strategies for promoting ferroptosis, particularly in cancer. Quercetin-induced TFEB activation promotes lysosomal ferritin degradation and thereby enhances ferroptotic susceptibility in cancer cells [97]. Fe_3_O_4_-NPs are internalized through endocytosis and subsequently accumulate in lysosomes, where their degradation releases redox-active iron and enhances ferroptosis [51]. More recently, Cañeque et al. designed fentomycin-1, a small-molecule activator of lysosomal iron that induces phospholipid oxidation and ultimately ferroptosis [114]. Their study further identified lysosomes as an early subcellular site of ferroptotic lipid peroxidation [114]. Ironomycin, a synthetic derivative of salinomycin, accumulates and sequesters iron within lysosomes. The resulting depletion of cytosolic iron promotes ferritin degradation, further increasing lysosomal iron accumulation, ROS production, and lysosomal membrane permeabilization, thereby inducing ferroptosis in breast cancer stem cells [138]. Together, these agents illustrate two major therapeutic approaches: enhancing lysosomal ferritin turnover and directly increasing the lysosomal pool of redox-active iron.

### 6.2. Translational Limitations and Future Directions

Despite this preclinical promise, the clinical translation of lysosome-targeted ferroptosis strategies remains challenging. First, the therapeutic effects of targeting lysosomal pathways may vary across tumor types and cellular states because of differences in lysosomal iron availability and molecular context, as illustrated by the context-dependent role of STEAP3 discussed above. For example, Fento-1 preferentially induces ferroptosis in iron-rich cluster of differentiation 44 (CD44) high-cancer-cell subpopulations, suggesting that redox-active lysosomal iron and CD44-dependent iron uptake may influence therapeutic sensitivity [114]. Second, lysosome-directed iron activation may cause off-target oxidative injury in normal cells and tissues. Fe_3_O_4_-NPs have been shown to reduce macrophage viability and activate p53-associated ferroptosis, highlighting their potential immunotoxicity [54]. In a separate in vitro blood–brain barrier model, pollution-mimicking Fe_3_O_4_-NPs were internalized by primary rat brain microvascular endothelial cells, crossed the endothelial monolayer, and caused cellular damage [53]. Although the formulations and exposure conditions in these studies differ from those used in therapeutic nanomaterials, the findings emphasize the need to carefully evaluate off-target toxicity. Third, nanoparticle size and formulation characteristics can substantially influence tumor accumulation, intratumoral distribution, iron release, therapeutic efficacy, and toxicity [55]. Tumor-targeted and stimulus-responsive delivery systems may therefore be required to improve spatial control and widen the therapeutic window.

## 7. Conclusions and Perspectives

**Figure 9:**
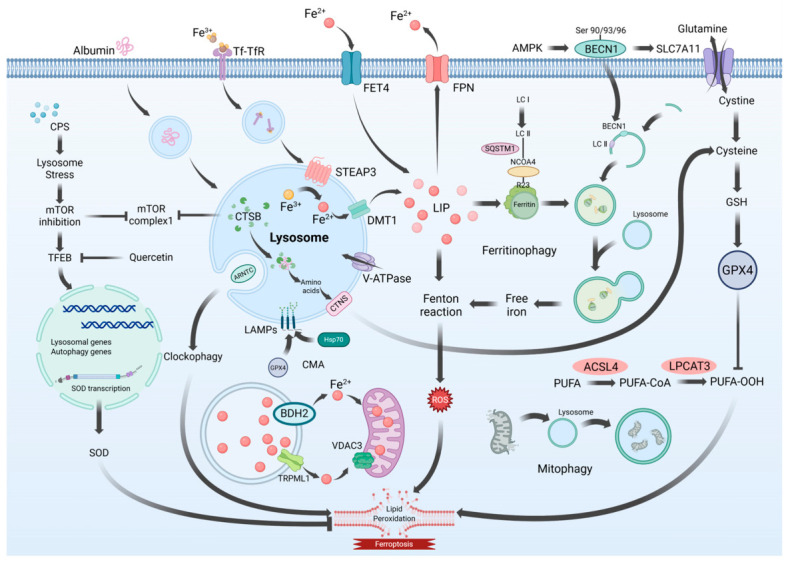
Lysosome-centered regulation of ferroptosis. This figure illustrates how lysosomes integrate autophagy, iron mobilization, lipid peroxidation, and antioxidant remodeling to control ferroptotic cell fate. Ferritinophagy promotes lysosomal iron release, whereas chaperone-mediated autophagy and clockophagy regulate ferroptosis-related protein turnover and lipid peroxidation pathways. Lysosome-derived Fe^2+^ expands the LIP and fuels Fenton reactions. In parallel, lysosomal modulation of the System Xc−GSH-GPX4 axis, cysteine salvage, and mTOR-TFEB signaling reshapes cellular redox homeostasis. Together, these coordinated pathways establish lysosomes as central hubs governing ferroptosis. ACSL4, acyl-CoA synthetase long-chain family member 4; AMPK, AMP-activated protein kinase; BECN1, Beclin 1; CMA, chaperone-mediated autophagy; DMT1, divalent metal transporter 1; GPX4, glutathione peroxidase 4; LIP, labile iron pool; mTOR, mechanistic target of rapamycin; NCOA4, nuclear receptor coactivator 4; ROS, reactive oxygen species; SQSTM1, sequestosome 1; TFEB, transcription factor EB. Created in BioRender. Luo, T. (2026) https://BioRender.com/7wb18h0 (accessed on 1 July 2026).

Over the past decade, lysosomes have emerged as central regulators of ferroptosis, extending their classical degradative role to active control of iron availability, redox balance, and lipid peroxidation. By coordinating iron trafficking, ferritin turnover, lysosome-dependent metabolic signaling, and crosstalk with mitochondria, lysosomes establish a critical intracellular platform that defines ferroptotic vulnerability. This integrative perspective underscores the importance of lysosomal function in both the initiation and modulation of ferroptosis across diverse pathological contexts, as summarized in Figure 9.

Lysosomal iron flux is not controlled by a single transporter. Instead, multiple lysosomal proteins, including TfR1, V-ATPase, STEAP3, DMT1, and LAMPs, operate in a coordinated or hierarchical manner during ferroptosis. Despite these advances, the mechanistic framework linking lysosomes to ferroptosis remains incomplete. While ferritinophagy-mediated iron release is widely recognized as a pro-ferroptotic mechanism, it remains unclear whether lysosome-derived iron primarily promotes ferroptosis after export into the cytosol or through localized oxidative reactions within the lysosomal lumen.

The dynamic regulation of lysosomal pH adds another layer of complexity. Acidification promotes ferritin degradation and iron mobilization, facilitating ferroptosis. In contrast, lysosomal alkalization may sequester redox-active iron within the organelle, limiting cytosolic ferroptotic responses while exacerbating lysosomal damage. Furthermore, the mechanisms by which cells selectively engage ferritinophagy over bulk autophagy during ferroptosis remain poorly understood, highlighting an important consideration for targeted therapeutic strategies.

BECN1-mediated inhibition of SLC7A11 occurs in an AMPK-dependent manner. However, the exact subcellular site of this interaction and its timing relative to ferroptosis initiation and amplification remain unclear. Additionally, the temporal and network-level coordination between lysosome-mediated regulation of the System Xc^−^–GSH–GPX4 axis and parallel antioxidant systems, including the thioredoxin and peroxiredoxin pathways, remains incompletely characterized. Clarifying these multilayered interactions will be essential not only for understanding lysosome-centered redox regulation but also for identifying context-specific therapeutic targets, predictive biomarkers, and rational combination strategies for ferroptosis-related diseases.

The pathological consequences of lysosome-dependent ferroptosis are also highly context-dependent. Lysosomal cysteine recycling and nutrient sensing can support ferroptosis resistance in cancer, whereas abnormal lipid catabolism, iron trapping, and excessive ferritinophagy can promote ferroptotic injury in neurodegenerative, cardiovascular, inflammatory, and ischemic diseases. Accordingly, lysosome-targeting small molecules and nanomaterials have shown preclinical therapeutic potential, but their clinical translation will require disease- and cell-type-specific stratification, improved spatial targeting, and careful evaluation of off-target oxidative toxicity.

Mathematical and computational models provide a useful framework for addressing this complexity. One systems-biology study incorporated ACSL4, stearoyl-CoA desaturase 1 (SCD1), ferroportin, transferrin receptor, and p53 into a mathematical model to classify cellular states as having high or low ferroptosis sensitivity [139]. Another study developed a Boolean model integrating p53, non-coding RNAs, Myc, xCT, and the GSH/GPX4 antioxidant pathway to predict ferroptosis, apoptosis, senescence, and drug resistance [140]. These models illustrate how network-level interactions can reveal regulatory relationships and potential combination targets that are difficult to identify from individual pathways alone. However, current models rarely represent lysosomes as distinct iron and redox compartments. Incorporating lysosomal iron, pH, ferritinophagy, TFEB signaling, and lysosomal membrane permeabilization may therefore improve the prediction of ferroptotic responses and therapeutic sensitivity.

## Figures and Tables

**Table 1:** Mechanistic involvement of LAMP isoforms in ferroptosis regulation.

Isoforms	Primary Mechanism	Role in Ferroptosis
LAMP-1	Transcriptional target of TFEB; Canonical lysosomal marker	Mediates ferritinophagy and promotes lysosomal iron release
LAMP-2A	CMA-specific receptor	Directly facilitates GPX4 degradation
LAMP-2B	Autophagosome-lysosome fusion	Participates in macroautophagy and indirectly regulates iron metabolism
LAMP-2C	RNautophagy/DNautophagy	Mediates nucleic acid autophagy and influences the expression of ferroptosis-related genes

CMA, chaperone-mediated autophagy; GPX4, glutathione peroxidase 4; TFEB, transcription factor EB.

**Table 2:** Representative roles of lysosome-dependent ferroptosis in human diseases.

Disease or Pathological Condition	Key Lysosome-Dependent Mechanism	Effect on Ferroptosis and Disease	Reference
Multiple solid tumors	Lysosomal degradation of extracellular cysteine-rich proteins supplies cysteine for GSH synthesis	Suppresses ferroptosis and supports tumor-cell survival under nutrient-limited conditions	[16]
Renal cell carcinoma	Lysosomal cystine dynamics regulate the AhR–ATF4 adaptive response	Promotes adaptation to cysteine limitation and reduces ferroptotic sensitivity	[131]
Neurodegeneration-related neuronal vulnerability	PSAP loss impairs lysosomal glycosphingolipid catabolism and promotes lipofuscin-associated iron trapping	Increases oxidative stress and neuronal ferroptosis	[132]
Multiple system atrophy	Pathological α-synuclein stabilizes NCOA4 and enhances FTH1 degradation	Promotes iron accumulation and oligodendrocyte ferroptosis	[133]
Atherosclerosis	DPP4 promotes NCOA4-dependent ferritinophagy and FTH1 destabilization	Induces endothelial ferroptosis and contributes to atherosclerotic progression	[134]
Heart failure	NAT10–SOAT1 signaling increases NCOA4 stability and ferritinophagy	Promotes cardiomyocyte ferroptosis and aggravates cardiac remodeling	[135]
Sepsis	STING interacts with NCOA4 to promote ferritinophagy in macrophages	Enhances macrophage ferroptosis, inflammation, and multiple-organ injury	[136]
Ischemic acute kidney injury	STING promotes NCOA4-mediated ferritinophagy in renal tubular cells	Increases iron-dependent lipid peroxidation and renal injury	[137]

AhR, aryl hydrocarbon receptor; ATF4, activating transcription factor 4; DPP4, dipeptidyl peptidase 4; FTH1, ferritin heavy chain 1; GSH, glutathione; NAT10, N-acetyltransferase 10; NCOA4, nuclear receptor coactivator 4; PSAP, prosaposin; SOAT1, sterol O-acyltransferase 1; STING, stimulator of interferon genes.

## Data Availability

No new data were created or analyzed in this study.

## Abbreviations

The following abbreviations are used in this manuscript:

AA	arachidonic acid
ACSL4	acyl-CoA synthetase long-chain family member 4
AhR	aryl hydrocarbon receptor
ALOX15	arachidonate lipoxygenase 15
AMPK	AMP-activated protein kinase
ARNTL	aryl hydrocarbon receptor nuclear translocator-like protein 1
ASK1	apoptosis signal-regulating kinase 1
ATF4	activating transcription factor 4
BDH2	3-hydroxybutyrate dehydrogenase 2
BECN1	Beclin 1
CD44	cluster of differentiation 44
CMA	chaperone-mediated autophagy
DMT1	divalent metal transporter 1
DPP4	dipeptidyl peptidase 4
DRP1	dynamin-related protein 1
ESCRT	endosomal sorting complexes required for transport
FIS1	mitochondrial fission 1 protein
FPN	ferroportin
FTL	ferritin light chain
FTH	ferritin heavy chain
GPX4	glutathione peroxidase 4
GSH	glutathione
IKKβ	inhibitor of nuclear factor κB kinase subunit β
IRE	iron-responsive element
IRP	iron regulatory protein
KEAP1	Kelch-like ECH-associated protein 1
LAMP	lysosome-associated membrane protein
LIP	labile iron pool
LMP	lysosomal membrane permeabilization
MEKK1	MAPK (mitogen-activated protein kinase)/ERK (extracellular-signal-regulated kinase) kinase kinase 1
mTOR	mechanistic target of rapamycin
mTORC1	mechanistic target of rapamycin complex 1
NAT10	N-acetyltransferase 10
NCOA4	nuclear receptor coactivator 4
NF-κB	nuclear factor κB
NRF2	nuclear factor erythroid 2-related factor 2
PLOOH	phospholipid hydroperoxides
PKCβII	protein kinase C βII
PSAP	prosaposin
PUFA	polyunsaturated fatty acid
ROS	reactive oxygen species
SCD1	stearoyl-CoA desaturase 1
SLC7A11	solute carrier family 7 member 11
SLC11A2	solute carrier family 11 member 2
SNARE	soluble N-ethylmaleimide-sensitive factor attachment protein receptor
SOAT1	sterol O-acyltransferase 1
SOD	superoxide dismutase
SQSTM1	sequestosome 1
STEAP3	six-transmembrane epithelial antigen of prostate 3
STING	stimulator of interferon genes
TCF25	transcription factor 25
Tf	transferrin
TFEB	transcription factor EB
TfR1	transferrin receptor 1
TRPML1	transient receptor potential mucolipin 1
TXNRD1	thioredoxin reductase 1
USP8	ubiquitin-specific peptidase 8
V-ATPase	vacuolar-type ATPase
VDAC1	voltage-dependent anion channel 1
VDAC3	voltage-dependent anion channel 3
xCT	cystine/glutamate antiporter light chain
